# ANGPTL8: An Important Regulator in Metabolic Disorders

**DOI:** 10.3389/fendo.2018.00169

**Published:** 2018-04-17

**Authors:** Mengdie Luo, Daoquan Peng

**Affiliations:** Department of Cardiovascular Medicine, The Second Xiangya Hospital, Central South University, Changsha, China

**Keywords:** angiopoietin-like protein 8, diabetes mellitus, ANGPTL3, triglyceride, diet

## Abstract

Long-term controversy regarding the role of angiopoietin-like protein 8 (ANGPTL8) in beta-cell proliferation and diabetes progression made it a research spotlight. Recently, the controversy was resolved. Although ANGPTL8 could not control beta-cell expansion and islet function, ANGPTL8 was still considered as a novel but atypical member in the ANGPTL family because of its unique structure and crucial effects on lipid metabolism. Besides, ANGPTL8 also participated in some other disorders such as non-alcoholic fatty liver disease and renal dysfunction. Understanding the features of ANGPTL8 may offer new diagnostic and therapeutic approaches to metabolic-related diseases. Therefore, we reviewed most recent findings about ANGPTL8 and aimed to provide an integrated picture of ANGPTL8.

## Introduction

Angiopoietin-like proteins (ANGPTLs) are a group of secreted glycoproteins composed of eight members (ANGPTL1–8) (1). ANGPTL1–7 share common structures and carry distinct functions (1). ANGPTL3 and ANGPTL4, highly homologous with each other, work in concert to regulate lipid metabolism in different nutritional states (2, 3). ANGPTL8, a 22-kDa protein with 198 amino acids, is a distinctive member in the ANGPTL family because of lacking the common structures shared by ANGPTL1–7 (Table 1) (2, 3). On the other hand, ANGPTL8 states its kinship by showing structural homology with the N-terminal domains of ANGPTL3 and ANGPTL4 (2, 3) and exhibiting functional similarity with these two proteins—binding with lipoprotein lipase (LPL) and regulating triglyceride metabolism (4) (Table 1). Therefore, ANGPTL8 has been considered as a novel but atypical member in the ANGPTL family (2, 3) and an emerging player in lipid metabolism (2, 3, 5). Besides, the long-term controversy regarding the role of ANGPTL8 in beta-cell proliferation (6) has intrigued many researchers to investigate the effects of ANGPTL8 on beta-cell expansion and islet function. No convincing evidence could be found to support the direct effects of ANGPTL8 on beta-cell replication (7–9), and thus ANGPTL8 is not regarded as a possible target for diabetes intervention. However, many epidemiological studies have demonstrated the alteration of ANGPTL8 concentration in metabolic diseases including diabetes, obesity, and metabolic syndrome. The relationship between ANGPTL8 and other biomarkers of these diseases has also been reported. Although results from these studies are inconsistent and still await clarification (6), these results indicate that ANGPTL8 may play a role in the disease emergence and progression. Regulating ANGPTL8 expression may become a novel pathway to normalize the disorders of lipid and glucose metabolism. In addition, in other diseases, such as polycystic ovary syndrome (PCOS) (10), adriamycin cardiomyopathy (11), and renal dysfunction (12), ANGPTL8 was also identified as a relevant modulator, which suggested additional functions of ANGPTL8 beyond the impacts on lipid and glucose metabolism. This review aims to provide an integrated picture of ANGPTL8 expression, regulation, and function, with special emphasis on its role in lipid and glucose metabolism. ANGPTL8, encoded by Gm6484 gene in mice (6) and C19orf80 gene in humans (13), is also known as lipasin, refeeding-induced in fat and liver, betatrophin, hepatocellular carcinoma-associated protein TD26, LOC55908 (6). In this review, the name “ANGPTL8” was adopted.

**Table 1:** Structure feature of ANGPTL8.

	ANGPTL8	ANGPTL1–7
Fibrinogen-like domain	✗	✓
Coiled-coil domain	✗	✓
Disulfide bond	✗	✓
Glycosylation	✗	✓
Signal peptide	✓	✓
SE1 segment	✓	ANGPTL3 and 4 (✓)

*ANGPTL, angiopoietin-like protein; SE1 segment, a segment that mediates lipoprotein lipase binding*.

## Mechanisms Underlying the Regulation of ANGPTL8 Expression

**Figure 1:**
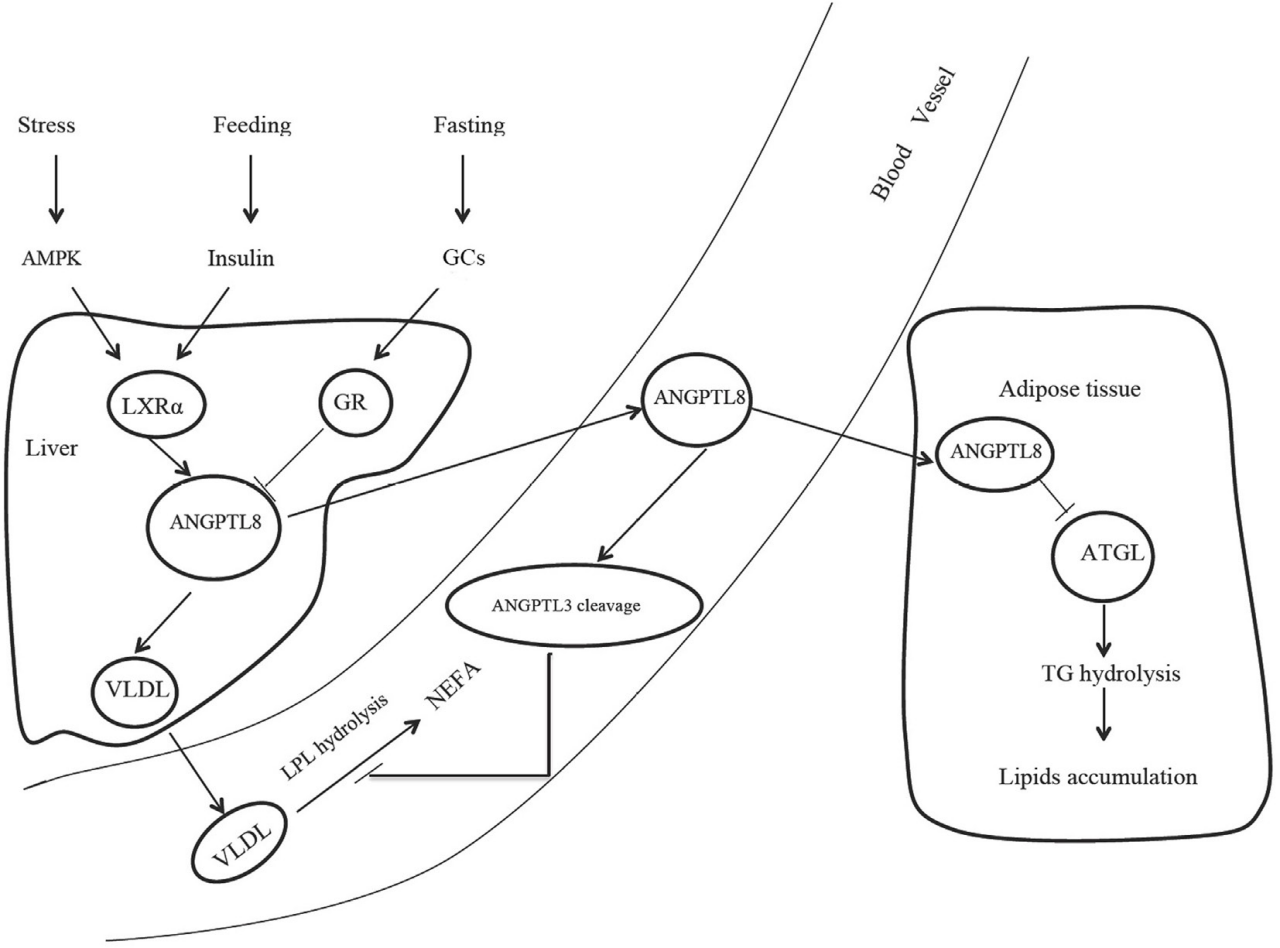
The role of ANGPTL8 in lipids metabolism. AMPK, adenosine monophosphate-activated protein kinase; GCs, glucocorticoids; LXRα, liver X receptor α; GR, glucocorticoid receptor; ANGPTL8, angiopoietin-like protein 8; VLDL, very low-density lipoprotein; LPL, lipoprotein lipase; NEFA, nonesterified fatty acid; ANGPTL3, angiopoietin-like protein 3; ATGL, adipose triglyceride lipase; TG, triglyceride.

The source of ANGPTL8 differs among species. Human ANGPTL8 is liver specific, while the mouse ANGPTL8 is enriched in the liver and fat tissue, including brown adipose tissue (BAT) and white adipose tissue (WAT) (2). Several mechanisms were found to be involved in the regulation of ANGPTL8 expression. Nutritional regulation plays an important role in the ANGPTL8 expression. Mice fed with high-fat diets exhibited significantly increased ANGPTL8 expression in the liver and BAT, while fasting treatment decreased ANGPTL8 expression by almost 80% in BAT and WAT (2). Dang et al. also observed that hepatic ANGPTL8 expression was oscillated in a circadian pattern and synchronized to food availability (14). The short half-life of ANGPTL8 mRNA (15.71 min) and the relatively long half-life of ANGPTL8 protein (2.47 h) guaranteed a rapid response to fasting/feeding transitions and a stable capability to exert corresponding functions after secretion (14). The effects of nutritional status on ANGPTL8 expression were also observed in human studies. In obese subjects with the metabolic syndrome prescribed to hypocaloric diets, ANGPTL8 level was inversely correlated with protein intake, particularly with animal-derived protein intake (15). Therefore, diet, especially the proportion of protein intake, was regarded as an important modulator in ANGPTL8 expression. Postprandial state activates liver X receptor α and then induces ANGPTL8 expression *via* insulin signaling (Figure 1). In adipocytes, insulin treatment could increase ANGPTL8 gene transcription by nearly 35-fold in a time- and dose-dependent manner (16). However, although ANGPTL8 gene did not respond to glucose treatment, insulin exerts its function on ANGPTL8 gene in case of glucose addition (16). Stress could also activate AMPK pathway and induce the response of liver X receptor α, leading to the increased ANGPTL8 expression (Figure 1). Results from cell models revealed that, an artificial stressor—histidine deprivation in the culture medium activated the RAS/RAF/MAPK signaling pathway and induced ANGPTL8 expression (17, 18). However, no research regarding the relationship between stress and ANGPTL8 expression in animal model and human was reported. In addition, inflammation might also participate in the ANGPTL8 regulation. *In vitro* experiments showed that tumor necrosis factor α treatment significantly decreased ANGPTL8 expression (16). A clinical study targeted at MS subjects found that circulating ANGPTL8 was positively correlated with high sensitivity C reactive protein (19), a typical inflammatory marker. At the same time, animal experiments indicate that ANGPTL8 may also be thermoregulated, because cold exposure could induce ANGPTL8 expression in BAT for more than three folds in mice (3). However, the detailed mechanism for the thermoregulation is still unclear.

## ANGPTL8 and Lipid Metabolism

Many studies have demonstrated that ANGPTL8 is a crucial modulator in lipid metabolism. A nonsynonymous sequence variation in ANGPTL8 gene (rs2278426) caused an arginine to tryptophan substitution at position 59 (R59W) in ANGPTL8 protein and contributed to lower plasma high density lipoprotein-cholesterol (HDL-C) in European Americans, Hispanics, and African-Americans (4, 20). The negative relationship between ANGPTL8 and HDL-C was also validated by a case–control study in Chinese subjects (21). These studies provided some clarification regarding the effects of ANGPTL8 on HDL-C; however, more direct evidences are still needed.

Although the relationship between ANGPTL8 and HDL-C called for more investigation, a deep insight into the role of ANGPTL8 in triglyceride metabolism was gained. Many population studies showed that circulating ANGPTL8 was positively correlated with triglyceride (22, 23), and findings from animal experiments supported the positive correlation of ANGPTL8 with triglyceride. Therefore, it is plausible that ANGPTL8 was inversely correlated with HDL-C because of the common opposite change between triglyceride and HDL-C. In mice, ANGPTL8 overexpression increased triglyceride levels for more than five folds (2), while ANGPTL8 deficiency reduced triglyceride by nearly two folds (24). The effects of ANGPTL8 on triglyceride metabolism were tightly associated with the presence of ANGPTL3, as reported by Quagliarini et al. found that ANGPTL8 lost impacts on triglyceride metabolism in ANGPTL3 knockout mice (4). ANGPTL8 promoted ANGPTL3 cleavage and bounded to the N-terminal of ANGPTL3 (4). ANGPTL8 and the N-terminal of ANGPTL3 formed a complex, and the complex orchestrated the inhibition of LPL and triglyceride modulation (4). In addition, Zhang et al. injected mice with ANGPTL8 monoclonal antibody and found that ANGPTL8 deficiency increased postprandial LPL activity specifically in cardiac and skeletal muscles (25). Based on these findings, Zhang et al. proposed a potential mechanism to demonstrate the effects of ANGPTL8 on triglyceride metabolism in different nutritional states (25, 26). In this model, ANGPTL3, ANGPTL4, and ANGPTL8 coordinated to regulate triglyceride trafficking (26). Food intake induced ANGPTL8 expression but suppressed ANGPTL4 expression. Subsequently, LPL activity in muscles controlled by ANGPTL3 and ANGPTL8 was inhibited, but LPL in WAT controlled by ANGPTL4 was activated. In this context, triglyceride was directed to WAT for degradation and storage. Conversely, fasting induced ANGPTL4 expression but suppressed ANGPTL8 expression, thereby leading to LPL activation in muscles and triglyceride mobilization to muscles for oxidation and energy supply.

Interestingly, some innovative studies found that ANGPTL8 was associated with adipogenesis and autophagy, indicating that ANGPTL8 might exert alternative functions independent of the regulation of LPL activity. ANGPTL8 could activate ERK signaling pathway and then induce Egr1 expression. Following these steps, adipose triglyceride lipase (ATGL), a crucial enzyme involved in triglyceride hydrolysis in adipocytes, was downregulated and lipid was accumulated in adipose tissue (17). On the other hand, ANGPTL8 knockdown significantly decreased lipid content in adipocytes during adipogenesis (16). In multiple *in vitro* adipogenesis models, the onset of lipid accumulation or lipolysis was corresponding to the increase or decrease of ANGPTL8 expression (16). The effect of ANGPTL8 on lipid metabolism on adipocytes seems dependent on the type of adipose tissue. For example, Martinez-Perez et al. found that BAT could produce ANGPTL8, and in turn ANGPTL8 induced WAT browning (27). The WAT browning was partly due to the fact that ANGPTL8 could promote the autolysosome maturation and the activation of autophagy (13). The effect of ANGPTL8 on autophagy explained how ANGPTL8 resulted in the degradation and catabolism of lipid droplets (13). The seemingly contradictory illustrations indicate that ANGPTL8 may work as a multifaceted player and present different functions under different conditions.

## ANGPTL8 and Glucose Metabolism

Recently, the debate about the role of ANGPTL8 in pancreatic beta-cell proliferation has been settled. The conclusion that ANGPTL8 did not control beta-cell expansion in the mouse model received unanimous agreement (7, 8). Consistent with this, plasma glucose and insulin concentration in different nutritional states did not present significant difference between wild-type mice and ANGPTL8 knockout mice (24). However, findings from population studies were controversial. Results on the comparison of ANGPTL8 concentration between controls and diabetes were inconsistent; presenting increased (21, 28–37), decreased (38), or unchanged (39, 40) (Table 2). The association of ANGPTL8 with insulin resistance (32, 33, 41), glucose (32, 34), C-peptide, and glycosylated hemoglobin (HbA1C) (37) were also contentious.

**Table 2:** ANGPTL8 concentration in diseases.

Diseases	ANGPTL8 concentration change	Kits manufacturer
T1DM	↑(28, 29)	EIAAB
T2DM	↑(21, 29–33, 35, 37)	EIAAB
	↑(34, 36)	Phoenix
	↓(38)	Cusabio
	–(39)	EIAAB
	–(40)	Aviscera
Obesity	↑(22)	EIAAB
	↑(36)	Phoenix
	↓(38, 42, 43)	Cusabio
	–(40)	Aviscera
MS	↑(19)	EIAAB
	↓(44)	Cusabio
NAFLD	↑(45)	Phoenix
Pregnancy	↑(27, 46)	EIAAB
GDM	↓(27)	EIAAB
	↑(46)	EIAAB
	↑(47)	USCN Life
	↑(48)	Phoenix
PCOS	↑(10)	Cusabio

*T1DM, type 1 diabetes mellitus; T2DM, type 2 diabetes mellitus; MS, metabolic syndrome; NAFLD, non-alcoholic fatty liver disease; GDM, gestational diabetes mellitus; PCOS, polycystic ovary syndrome*.

A study separated patients suffering from type 2 diabetes mellitus (T2DM) into normoalbuminuria, microalbuminuria, and macroalbuminuria groups according to albumin/creatinine ratio and found that elevated ANGPTL8 was associated with increased albuminuria and higher risk for diabetic nephropathy (OR = 5.65, 95% CI 2.17–4.57, *P* < 0.001) (12). The relationship between ANGPTL8 and biomarkers of renal function was also observed in studies focusing on dyslipidemia (49) and MS (23). Since renal dysfunction influenced lipid metabolism, the relationship between ANGPTL8 and dyslipidemia might be confounded by the degree of proteinuria.

In addition, plasma ANGPTL8 was increased approximately 10 folds during gestational period compared to non-pregnant women (27, 46) and decreased with the progression of pregnancy (27), returning to the normal range in postpartum period. Compared to healthy controls, subjects with gestational diabetes mellitus present increased ANGPTL8 in maternal and cord blood (47, 48).

Results from these studies present a wide range of variations (Table 2). Alternative explanations for discrepancies among different research are as follows. First, one possible explanation for the discrepancy in the results from different studies could be the adoption of different ELISA kits (50). ANGPTL8 was hypothesized to be cleaved *in vivo* and to release C-terminal fragments. Kits manufactured by EIAAB (Catalogue No. E1164H; Wuhan, Hubei, China) recognize N-terminus of ANGPTL8 and measure the full-length protein, while kits manufactured by Phoenix Pharmaceuticals (Catalogue No. EK-051-55; Burlingame, CA, USA) recognize C-terminus of ANGPTL8 and measure both full-length protein and C-terminal fragments. Therefore, ANGPTL8 level measured by the latter one is higher than that measured by the former one. Although independent studies showed correlations between results generated using these two ELISA kits [*r* = 0.559, *P* < 0.001 (12); *r* = 0.760, *P* < 0.001 (46)], the difference observed suggested that caution should be exercised regarding the interpretation of the results. As for ELISA kits made by other manufacturers (38, 40, 47), no definite information could be found regarding their specifications. In addition, vitamin D status may also account for the inconsistent results (23). A cross-sectional study divided subjects into different groups, i.e., the group with vitamin D deficiency and the group with high quartile of vitamin D, and assessed the associations of ANGPTL8 and cardiometabolic variables. The study found that the associations between ANGPTL8 and metabolic profiles were opposite in these two groups (23), implying that vitamin D status and other factors undiscovered yet might interfere with the observation.

## ANGPTL8 in Metabolic and Non-Metabolic Diseases

In addition to the lipid and glucose metabolism, ANGPTL8 has been reported to be involved in many other disorders. Alterations in ANGPTL8 level were found in subjects with obesity (22, 36, 38, 40, 42, 43), but the pattern of alteration was controversial (Table 2). Early report by Maurer et al. found that weight loss could increase ANGPTL8 expression (51), but changes in response to weight loss seemed subject to the method of weight loss. For example, one study showed that surgery-induced weight loss increased ANGPTL8 expression while diet-induced weight loss did not (52). Besides, ANGPTL8 increment in 1 month after bariatric surgery was a valuable predictor of T2DM remission in the 1 year follow-up (OR: 1.870, 95% CI 1.152–2.035, *P* = 0.011) (53).

Studies showed that ANGPTL8 was positively correlated with the incidence of non-alcoholic fatty liver disease (NAFLD) and significantly elevated in mice and humans with NAFLD (45). ANGPTL8 elevation may be related to endoplasmic reticulum stress, an important player in the NAFLD (45). Recent study indicated that elevated ANGPTL8 could predict higher risk of developing PCOS (OR: 2.51, 95% CI 1.31–4.81, *P* = 0.006) (10). Another exploratory study has investigated the role of ANGPTL8 in myocardial regeneration. In this study, ultrasound-targeted microbubble destruction (UTMD) was employed to deliver human ANGPTL8 gene to the rats with adriamycin cardiomyopathy (11). UTMD-ANGPTL8 gene therapy activated a specific ANGPTL membrane receptor, paired immunoglobulin-like receptor B, and stimulated the proliferation of cardiac progenitor cells located at epicardium, accompanied by the restoration of cardiac function and reversal of established adriamycin cardiomyopathy (11).

## Perspective

During fasting state, elevated glucocorticoids activate glucocorticoid receptor and then suppress ANGPTL8 transcription. Postprandial state and stress response activate the response of liver X receptor α and ANGPTL8 expression *via* insulin signaling and AMPK pathway, respectively. Elevated ANGPTL8 increases plasma triglyceride concentration through two main mechanisms. First, ANGPTL8 could favor very low-density lipoprotein (VLDL) secretion in the liver. Second, ANGPTL8 could promote ANGPTL3 cleavage and inhibit LPL activity, thus decreasing VLDL hydrolysis. In adipose tissue, ANGPTL8 could suppress ATGL expression and reduce triglyceride hydrolysis (Figure 1). Although recent studies reported here have revealed a mechanistic connection between ANGPTL8 and lipid homeostasis, many questions still remain to be addressed.

(1) ANGPTL8 expression pattern in some pathological conditions such as obesity, diabetes, and MS remains unclear. Relationship between ANGPTL8 and biomarkers in these diseases is also vague. More strictly controlled nutritional status and technique to measure ANGPTL8 may help to resolve the problem.
(2) Many physiological and pathophysiological factors have been identified to regulate ANGPTL8 expression. However, the detailed underlying mechanisms are still unknown. Whether ANGPTL8 expression could be responsive to a wide variety of stressors or only to histidine deprivation remains to be clarified. In addition, how changes in temperature, insulin levels, and inflammation might influence ANGPTL8 expression and the associated signaling pathways remains to be determined.
(3) The collaboration between ANGPTL3 and ANGPTL8 warrants further investigation. In ANGPTL8 knockout mice, full-length ANGPTL3 would increase while N-terminal fragments decrease due to lack of ANGPTL8 cleavage as previously mentioned (4). However, immunoblot analysis indicated that ANGPTL8 was not the prerequisite for ANGPTL3 cleavage (4). In addition, contradicted with previous findings, in ANGPTL3 knockout mice, ANGPTL8 treatment decreased triglyceride, implying the alternative role of ANGPTL8 in triglyceride metabolism.
(4) The ANGPTL 3-4-8 model puts emphasis on the balance of ANGPTLs and LPL activity among different tissues. However, since ANGPTLs can be found in the circulation, the effects of local ANGPTLs and systemic ANGPTLs on LPL regulation should not be considered in isolation. The tissue-specific ANGPTL knockout animal models and systemic multiple-ANGPTLs knockout animal models may be helpful.
(5) ANGPTL8 monoclonal antibody was applied in mice to augment postprandial LPL activity and lower triglyceride (25). A fully human monoclonal antibody was developed and applied in monkeys. The ANGPTL8 blockade strongly reduced plasma triglyceride (54). ANGPTL8 inhibition may be a promising therapeutic avenue for the treatment of hypertriglyceridemia.
(6) ANGPTL8 was only detected in mammals and involved in some physiological process exclusive to mammals such as homeothermy and pregnancy (27). ANGPTL8 expression was also associated with the GDM status (48). Research regarding the effects of ANGPTL8 on thermogenic machinery and metabolic regulation during the fetal–neonatal transition may shed light on the approaches to neonatal hypothermia and metabolic disorders.

Our current knowledge on ANGPTL8 only represents the tip of the iceberg, and the remaining questions about ANGPTL8 call for further studies. Answers to these questions may provide a solid foundation for the application of ANGPTL8-targeted therapy in the future.

## Author Contributions

All authors participated in article drafting and revision.

## Conflict of Interest Statement

The authors declare that the research was conducted in the absence of any commercial or financial relationships that could be construed as a potential conflict of interest.
